# Prognostic factors in the treatment of gastric mucosal atypical hyperplasia by endoscopic submucosal dissection

**DOI:** 10.1186/s12893-022-01832-4

**Published:** 2022-11-07

**Authors:** Yongping Hong, Xingxing Chen, Guofeng Li

**Affiliations:** 1 Department of Anorectal Surgery, The First People’s Hospital of Xiaoshan District, Xiaoshan Affiliated Hospital of Wenzhou Medical University, No. 199 South Shixin Road, Hangzhou, 311200 Zhejiang People’s Republic of China; 2 Department of Ultrasound, The First People’s Hospital of Xiaoshan District, Hangzhou, 311200 Zhejiang People’s Republic of China

**Keywords:** Endoscopic submucosal dissection, Platelets, Albumin, Atypical hyperplasia, Prognosis

## Abstract

**Background:**

Endoscopic submucosal dissection (ESD) is becoming increasingly popular as a treatment for precancerous lesions and early cancers of the stomach. However, there have been few studies on the factors associated with the recurrence of precancerous lesions after ESD.

**Methods:**

To investigate the prognostic factors of gastric intraepithelial neoplasia, we retrospectively analyzed 115 patients who were treated with ESD between February 2018 and January 2020. Chi-square test and Fisher’s extract test were used to select factors for further investigation, and prognostic analysis was carried out with the Kaplan–Meier method and a Cox regression model.

**Results:**

Platelet counts (P = 0.027) and albumin levels (P = 0.011) were both lower in patients with recurrence than in patients without recurrence of gastric mucosal atypical hyperplasia after ESD.

**Conclusions:**

This study reveals that low platelet counts and albumin levels were probably unfavorable prognostic factors in mucosal atypical hyperplasia of the stomach.

## Introduction

Atypical hyperplasia presenting in the gastric mucosa is a type of precancerous lesion of gastric cancer. The results of a large Dutch cohort study of patients with a first diagnosis of precancerous gastric lesions showed that the annual incidence of gastric cancer was 0.1%, 0.25%, 0.6%, and 6% in the groups with atrophic gastritis, intestinal metaplasia, mild–moderate atypical hyperplasia, and severe atypical hyperplasia, respectively [1]. Another prospective study in Italy with a mean follow-up of 14 years showed a cancer rate of 8.9% and 68.7%, respectively, for mild–moderate, and severe atypical hyperplasia [2]. Therefore, in view of the above risks, the treatment of gastric atypical hyperplasia is very necessary. The positive detection rate of atypical hyperplasia has been rising as endoscopy has become more common. Endoscopic submucosal dissection (ESD) can be used to treat precancerous lesions and early cancers, and has the advantages of a large resection range, access to the whole specimen, and ease of pathological evaluation [3, 4]. Many studies have explored the prognosis of early gastric cancer after ESD treatment [5, 6]. A large follow-up study found that *H. pylori*, smoking and low levels of dietary vitamin C were associated with the risk of progression to gastric atypical hyperplasia [7]. However, there has been little research on the factors associated with the recurrence of gastric mucosal atypical hyperplasia after ESD. If the recurrence of atypical hyperplasia in the gastric mucosa can be reduced, the incidence of early gastric cancer will be reduced accordingly, which is of great clinical interest. Therefore, we conducted this study to explore the risk factors for atypical hyperplasia recurrence after ESD, aiming to make better recommendations and improve the prognosis for patients with the above diseases.

## Materials and methods

### Patients and study design

The inclusion criteria for patients were the following: (1) Total removal of the lesion through ESD; (2) atypical hyperplasia confirmed by postoperative pathology; and (3) negative pathological margins. The exclusion criteria were blood dyscrasias, systemic inflammatory disease, autoimmune disease, liver disease, renal disease, thyroid disease/cancer, and medical treatment with an anticoagulant. Nine patients who were diagnosed with gastric cancer including carcinoma in situ were also excluded, and 10 patients were lost to follow-up. Ultimately, 115 patients who received ESD treatment from February 2018 and January 2020 (48 months) at The First People’s Hospital of Xiaoshan District, China were included in this study.

All patients received ESD treatment from experienced digestive physicians. Intraoperative and postoperative complications such as bleeding and perforation received timely and effective treatment. All the lesions were completely resected, and the margins were all negative.

The follow-up time was from the day of ESD treatment until January 31, 2022. We required that included patients had at least 12 months of follow up and 2 or more endoscopic findings, or did not meet the above inclusion criteria but had pathological findings of atypical hyperplasia on endoscopy during follow-up. The median follow-up duration was 30 months. Most patients were followed up every 3 to 6 months with a gastroscopy review. We defined recurrence as the endoscopic pathology of the original lesion site of atypical hyperplasia in a subsequent review. The times to recurrence or death were analyzed as end events.

Because our study was retrospective and patients’ medical records were reviewed from February 2018 and January 2020, it was difficult to obtain patients’ informed consent. Since our research method had a very low risk, the Institutional Review Board of The First People’s Hospital of Xiaoshan District approved our research without requiring consent.

### Clinical characteristics and biochemical measurements

We first collected the clinical characteristics for all patients, including sex, age, lesion morphology and pathological grade, and then evaluated the biochemical indicators. Venous blood samples were taken from patients after a morning fast within 5 days before surgery. Whole blood samples were collected in a special blood collection tube, and all blood samples were processed in a short time. The white blood cell (WBC), neutrophil, lymphocyte, platelet and albumin levels and blood types were measured with a blood analyzer. The inter- and intra-assay coefficients of variation for all assays were less than 5%. We recorded the patients’ histopathological information, including the lesion morphology and degree, atrophic gastritis and intestinal metaplasia. Positive for *H. pylori* infection using histocytological Jimsa staining. We included bleeding and en bloc resection as variables, and en bloc resection was defined as the removal of the lesion completely with negative margins. Finally, we also collected information about the patient’s health, expressed in American Society of Anesthesiologists (ASA) score.

The neutrophil/lymphocyte ratio (NLR) was calculated as the absolute neutrophil count divided by the measured absolute lymphocyte count. The platelet/lymphocyte ratio (PLR) was calculated as the absolute platelet count divided by the measured absolute lymphocyte count. The ideal cut-off values for the NLR, PLR, platelet count, albumin level, etc. were obtained from their mean values.

### Statistical analysis

Baseline data were obtained from the electronic medical records of our hospital. Endoscopic pathology information that could not be reviewed in our hospital was obtained by telephone follow-up. Student’s t test was used to compare the non-categorical variables of the two groups. Chi-square tests and Fisher’s exact test were used to compare categorical variables and for the initial screening of recurrence factors, then the Kaplan–Meier method was used for further assessment of the recurrence rate. Variables with P values < 0.05 were included in a multivariate Cox hazards regression model with a regression elimination strategy. A two-tailed P-value < 0.05 was set as the statistical significance threshold. Statistical analyses were performed with SPSS Version 22.0 (SPSS Inc., Chicago, IL, USA).

## Results

In total, 115 patients were enrolled in our study, including 77 male patients and 38 female patients, and the median age was 61.0 ± 10.0 years (range 36–89). The patients’ lesions ranged from 5 to 50 mm in maximum diameter (14.9 ± 8.4 mm) and located on upper third of stomach in 9 patients, middle third in 14 patients and lower third in 92 patients. The rate of en bloc resection was 93.0% (107/115) and the unsuccessful en bloc resection rate was 7.0% (8/115). Six patients emerged bleeding during the procedure and all received effective hemostasis. Seven patients developed fever and 31 patients developed abdominal pain in the postoperative period. The median time to follow-up was 30 months, with a range of 3 to 48 months (interquartile range (IQR) = 26–37 months). During the follow-up period, 19 patients relapsed. In addition, in the pathology review, two patients were found to have atypical hyperplasia far from the primary site, but these were categorized into the non-recurrence group. Only one patient died, but the cause of death was unknown. The clinicopathological characteristics of the patients with and without recurrence are shown in Table 1.

**Table 1:** Baseline characteristics of the patients

Parameters	Recurrence (n = 19)	Recurrence-free (n = 96)	P value
Age (years)			0.89^*^
≤ 60	11 (57.9)	54(56.2)	
> 60	8 (42.1)	42(43.8)	
Gender			0.068^*^
Male	16 (84.2)	60 (62.5)	
Female	3 (15.8)	36 (37.5)	
Lesions size (cm)			0.216^*^
≥ 2	7 (36.8)	23 (24.0)	
< 2	12 (63.2)	73 (76.0)	
Location			0.109^**^
Upper 1/3	3 (15.8)	6 (6.3)	
Middle 1/3	4 (21.0)	10 (10.4)	
Lower 1/3	12 (63.2)	80 (83.3)	
Gross appearance			0.159^*^
Depressed	6 (31.6)	17 (17.7)	
Flat	5 (26.3)	47 (49.0)	
Elevated	8 (42.1)	32 (33.3)	
Pathological grade			0.241^*^
Mild–moderate	14 (73.7)	57 (59.4)	
Severe	5 (26.3)	39 (40.6)	
NLR			0.567^*^
≤ 1.97	13 (68.4)	59 (61.5)	
> 1.97	6 (31.6)	37 (38.5)	
PLR			0.512^*^
≤ 122.1	13 (68.4)	58 (60.4)	
> 122.1	6 (31.6)	38 (39.6)	
Platelets (× 10^9^/L)			0.027^*^
≤ 207.9	14 (73.7)	44 (45.8)	
> 207.9	5 (26.3)	52 (54.2)	
Albumin (g/L)			0.011^*^
≤ 40.8	15 (78.9)	45 (46.9)	
> 40.8	4 (21.1)	51 (53.1)	
WBC (× 10^9^/L)			0.29^*^
≤ 5.6	7 (36.8)	48 (50.0)	
> 5.6	12 (63.2)	48 (50.0)	
Bleeding			0.258^**^
Yes	17(89.5)	92 (95.8)	
No	2 (10.5)	4 (4.2)	
En bloc resection			0.125^**^
Yes	16(84.2)	91 (94.8)	
No	3 (15.8)	5 (5.2)	
*H. pylori* infection^a^			0.321^*^
Negative	3 (17.7)	27 (29.4)	
Positive	14 (82.3)	65 (70.6)	
Atrophic gastritis			0.243^*^
Negative	10(52.6)	64 (66.7)	
Positive	9 (47.4)	32 (33.3)	
Intestinal metaplasia			0.118^*^
Negative	8 (42.1)	59 (61.5)	
Positive	11(57.9)	37 (38.5)	
ASA score			0.447^*^
1	12(63.2)	69 (71.9)	
2	7 (36.8)	27 (28.1)	

Values are presented as mean ± SD or number (%)

*NLR* neutrophil-to-lymphocyte ratio, *PLR* platelet-to-lymphocyte ratio, *WBC* white blood cell, *ASA* American Society of Anesthesiologists

^*^Chi-square test. **Fisher’s extract test

^a^Exception is where pathologic report is absent

We next evaluated biochemical indicators in patients’ blood samples, and grouped patients accordingly. The platelet data were normally distributed (Kolmogorov–Smirnov test P = 0.95), the mean platelet count was 207.9 × 10^9^/L. Means can better represent the characteristics of the data, so we selected the mean platelet count (207.9 × 10^9^/L) as the cut-off value to divide the patients into two groups. Of the 115 patients, 57 had platelet counts less than 207.9 × 10^9^/L and 58 had platelet counts greater than 207.9 × 10^9^/L. The same method was applied to other non-categorical variables, including the WBC count, NLR, PLR and albumin level. There were no statistically significant differences between the recurrence and non-recurrence groups in terms of age, sex ratio, tumor morphology, pathological grade, NLR, PLR, WBC, bleeding, en bloc resection, *H. pylori*, atrophic gastritis, intestinal metaplasia and ASA score. However, the platelet counts (P = 0.027) and albumin levels (P = 0.011) differed significantly between patients with and without recurrence.

**Fig. 1:**
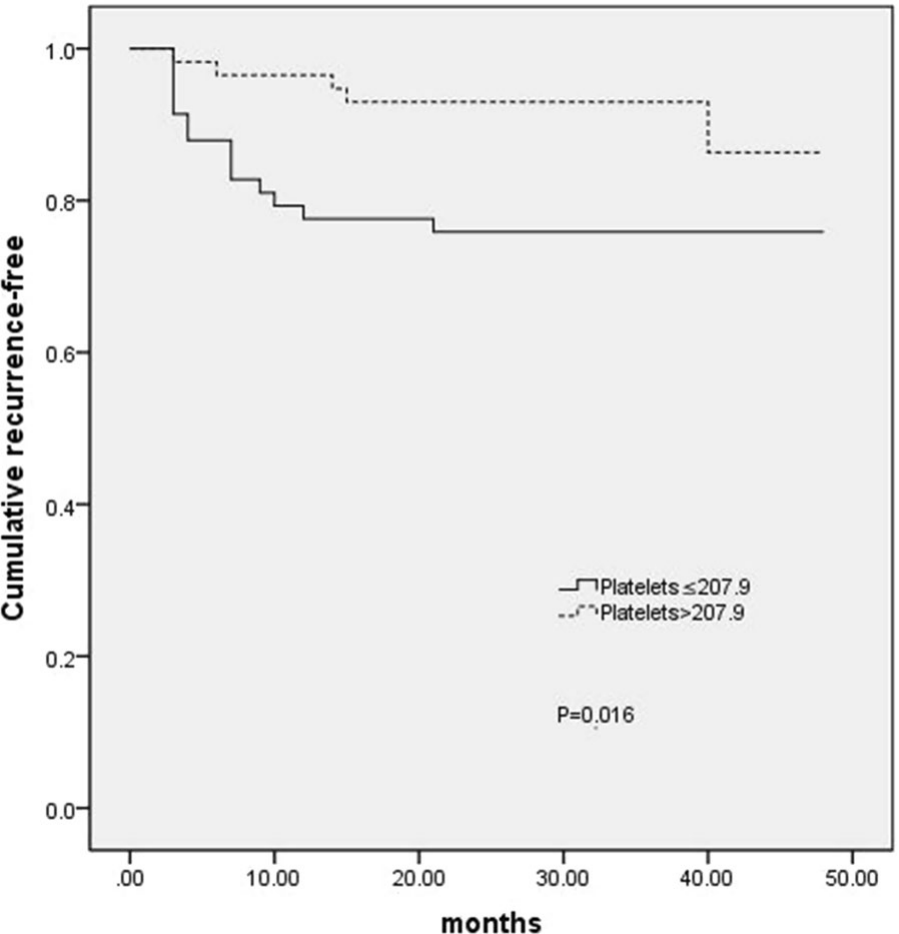
Kaplan–Meier analysis of platelet counts in gastric mucosal atypical hyperplasia patients

**Fig. 2:**
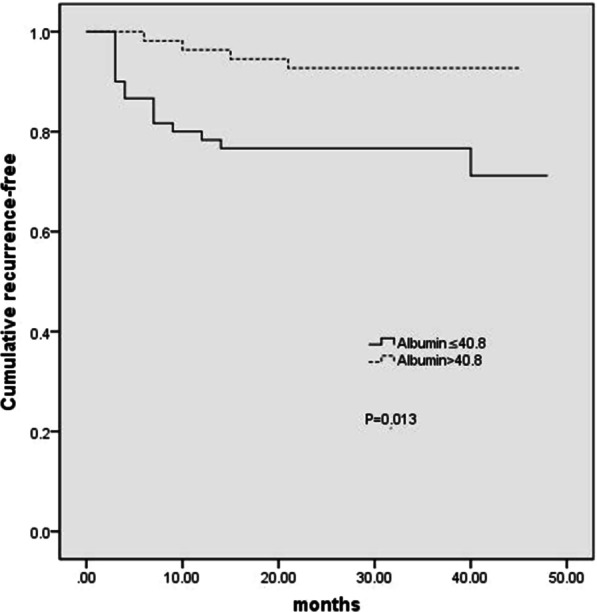
Kaplan–Meier analysis of albumin levels in gastric mucosal atypical hyperplasia patients

The recurrent lesions consisted of severe atypical hyperplasia (5 cases, 26.3%) and mild–moderate atypical hyperplasia (14 cases, 73.7%). Endoscopic findings in recurrent patients showed 5 lesions to be ulcerated, 8 lesions to be elevated and 6 lesions to be erosive. Of the patients who relapsed, two patients received a second ESD treatment after learning the results. The median time to recurrence was 7 months, with a range of 3 to 40 months (IQR = 3–12 months). The median number of endoscopies is 3 times, with a range of 1 to 6 times (IQR = 3–4 times). We used the Kaplan–Meier method to calculate the cumulative incidence of recurrence in the high-platelet and low-platelet groups. The cumulative incidence of recurrence was higher in the low-platelet group than in the high-platelet group (P = 0.016, Fig. 1). We also used the Kaplan–Meier method to analyze the cumulative incidence of recurrence according to albumin levels. The risk of recurrence was higher in the low-albumin group than in the high-albumin group (P = 0.013, Fig. 2). Finally, Cox factor regression analysis demonstrated that albumin levels and platelet counts were independent prognostic factors in gastric mucosal atypical hyperplasia patients (Table 2).

**Table 2:** Multivariate analysis of recurrence in patients with gastric mucosal atypical hyperplasia

	Hazard ratio	95% CI	P-value
Platelets	0.312	0.110–0.886	0.029
Albumin	0.286	0.094–0.866	0.027

## Discussion

This study demonstrated that albumin and platelet levels were probably the influential factors in the prognosis of gastric precancerous lesions after ESD. As indicated in the summary of patients’ baseline characteristics (Table 1), albumin and platelet levels differed substantially between the recurrence and recurrence-free groups. Specifically, low levels of platelets and albumin may increase the recurrence rate.

ESD was introduced primarily to promote en bloc resection of early gastrointestinal tumors, and has become more and more popular in Asia and the rest of the world [8]. Some studies have suggested that ESD can be considered a standard treatment for early gastric cancer, as it is associated with low incidences of lymph node metastasis and local recurrence [9–13]. A retrospective study of a large population indicated that the cumulative incidences of early gastric cancer after ESD treatment were 9.5%, 13.1% and 22.7% after 5, 7 and 10 years, respectively [14]. Platelet counts, lymph node metastasis, etc. may be prognostic factors for early gastric cancer, according to some previous studies [15, 16]. However, few studies have determined the factors associated with precancerous lesion recurrence after ESD. We investigated such factors in this study, and found that low platelet counts and albumin levels were associated with the recurrence of atypical hyperplasia after ESD.

Platelet counts are determined by the balance between platelet productivity and consumption. Depending on the effective compensation mechanism, normal platelet counts may mask highly hypercoagulative and proinflammatory cancer activity [17]. High platelet levels can lead to poor prognoses in various cancers, including pancreatic cancer, gastric cancer, colorectal cancer, endometrial cancer and ovarian cancer [18–22]. By responding to molecules that tumors produce to stimulate angiogenesis, platelets can cause thrombosis events that facilitate cancer progression [23]. However, based on the present data, we consider platelets to exert a positive effect in preventing the recurrence of gastric mucosal atypical hyperplasia.

Platelets may influence the prognosis of patients through some of their functions. Platelets are anucleate blood cells that break off from megakaryocytes in human bone marrow. They are greatly versatile effectors of hemostasis, immune activity and inflammation, and are involved in host defense, immune surveillance and response to injury. More than 300 proteins and lipids can be secreted or translocated to the plasma membrane by activated platelets. Platelet factor 4, a member of the CXC chemokine family, can promote monocyte differentiation to macrophages [24], which is essential for the innate immune response against *H. pylori* [25]. Various types of gastric mucosal cell injury can cause the formation of gastric precancerous lesions [26]. In the chronic process of gastric ulcer, as a form of mucosal injury, its edge can evolve into atypical hyperplasia [27]. Therefore helping the healing of gastric ulcer can reduce the incidence of atypical hyperplasia. One study reported that macrophages were helpful in the healing of gastric ulcers by promoting angiogenesis via upregulation of cyclooxygenase-2/prostaglandin E2 production [28]. On the other hand, platelets could release vascular endothelial growth factor to accelerate healing of gastric ulcers [29]. Nitric oxide (NO) and its derivatives such as OH^−^ and NO_2_^−^ could cause DNA damage and tissue injury leading to the formation of atypical hyperplasia or even cancer [30, 31]. Platelet-derived growth factor can inhibit macrophage production of nitric oxide to reduce cellular injury [32]. Thus, platelet-related mechanisms may have appealing effects on the outcome of gastric mucosal atypical hyperplasia.

Nutritional imbalances are among the causes of many diseases, and the serum albumin level is an indicator of an individual’s nutritional status. Hypoalbuminemia can cause delayed wound healing. Furthermore, there is a complex interaction between atypical hyperplasia and inflammation [33]. Serum albumin levels are associated with systemic inflammatory conditions; for instance, inflammatory factors such as IL-6 and IL-4 can affect the synthesis of albumin by hepatocytes and reduce serum albumin levels [34]. Therefore, low serum albumin levels may be associated with a poor prognosis in the present study.

From the above study, we hope to suggest better follow-up for patients after ESD, for example, closer follow-up of patients with relatively low platelets and relatively low albumin to detect their poor prognosis earlier.

There are some limitations to our research. This was a retrospective study with inevitable selection bias. In addition, the mechanism responsible for low platelet and albumin levels in patients with recurrent gastric mucosal atypical hyperplasia requires further exploration and clarification. In conclusion, our study revealed that low platelet and albumin levels were probably unfavorable prognostic factors in intraepithelial neoplasia of the stomach after ESD. Because of the small sample size included in our study, this may make the conclusions less evidential in strength. We will therefore collect a larger sample in the future to refine the study. The exact mechanism whereby low platelet and albumin levels are associated with precancerous lesions of gastric cancer will also be investigated in future studies.

## Data Availability

The datasets used and/or analysed during the current study available from the corresponding author on reasonable request.
